# Component-Resolved Diagnosis of American Cockroach (*Periplaneta americana*) Allergy in Patients From Different Geographical Areas

**DOI:** 10.3389/falgy.2021.691627

**Published:** 2021-06-29

**Authors:** Andrea Wangorsch, Annette Jamin, Stephanie Eichhorn, Isabel Pablos, Swati Sharma, Bettina Schweidler, Bianca Kastner, Sabrina Wildner, Joachim Saloga, Frank Führer, Reinaldo Rafael Reyna Orozco, Roya Sherkat, Somayeh Sadeghi, Fardis Teifoori, Jung-Won Park, Peter Briza, Stefan Vieths, Fatima Ferreira, Naveen Arora, Jonas Lidholm, Gabriele Gadermaier, Stephan Scheurer

**Affiliations:** ^1^Molecular Allergology, Paul-Ehrlich-Institut, Langen, Germany; ^2^Department of Biosciences, Paris Lodron University of Salzburg, Salzburg, Austria; ^3^Allergy and Immunology Section, CSIR-Institute of Genomics and Integrative Biology, New Delhi, India; ^4^Department of Dermatology, University Medical Center of the Johannes Gutenberg-University Mainz, Mainz, Germany; ^5^Batch Control and Allergen Analysis, Paul-Ehrlich-Institut, Langen, Germany; ^6^Clinical Immunology Service in Hospital Militar Dr. Carlos Arvelo, Caracas, Venezuela; ^7^Acquired Immunodeficiency Research Center, Isfahan University of Medical Sciences, Isfahan, Iran; ^8^Department of Internal Medicine, Yonsei University College of Medicine, Seoul, South Korea; ^9^Thermo Fisher Scientific, Immunodiagnostics, Uppsala, Sweden

**Keywords:** cockroach allergy, American cockroach, *Blattella germanica*, component-resolved diagnosis, *Periplaneta americana* (Insecta)

## Abstract

**Background:** Manifestation of respiratory allergy to American cockroach (*Periplaneta americana*) is prominent in the subtropical and tropical areas. However, co-existing perennial indoor inhalant allergies frequently compromise clinical diagnosis of cockroach allergy, and the analysis of sensitization pattern is limited by the lack of *Periplaneta* allergens widely available for component-resolved diagnostics (CRD).

**Objective:** To evaluate a collection of previously described recombinant *Periplaneta* allergens for CRD in cockroach allergy.

**Methods:** A panel of nine recombinant *Periplanet*a allergens (Per a 1–5, 7–10) was generated, purified, and subjected to physicochemical characterization by applying circular dichroism (CD) spectroscopy, dynamic light scattering (DLS), amino acid (AA) analysis, and mass spectrometry (MS). Patients (*n* = 117) from India, Korea, Venezuela, and Iran, reporting perennial respiratory indoor allergies with IgE sensitization to cockroach (*P. americana and/or Blattella germanica*), were included. The sensitization profile was monitored by the experimental ImmunoCAP testing.

**Results:** ImmunoCAP testing confirmed IgE sensitization to *Periplaneta* and/or *Blattella* extract in 98 of 117 patients (*r* = 0.95). Five out of 117 patients were sensitized to only one of the two cockroach species. Within the whole study group, the prevalence of sensitization to individual allergens varied from 4% (Per a 2) to 50% (Per a 9), with the highest IgE values to Per a 9. Patients from four countries displayed different sensitization profiles at which Per a 3 and Per a 9 were identified as major allergens in India and Korea. *Periplaneta*-derived lipocalin and myosin light chain were characterized as new minor allergens, designated as Per a 4 and Per a 8. *Periplaneta* extract showed higher diagnostic sensitivity than all individual components combined, suggesting the existence of allergens yet to be discovered.

**Conclusion:** Utilization of a panel of purified *Periplaneta* allergens revealed highly heterogeneous sensitization patterns and allowed the classification of lipocalin and myosin light chain from *Periplaneta* as new minor allergens.

## Introduction

The most common indoor aeroallergens are house dust and storage mites, pet dander, mold, and cockroaches (1). German cockroach (*Blattella germanica*), American cockroach (*Periplaneta americana*), Oriental cockroach (*Blatta orientalis*), brown-banded cockroach (*Supella longipalpa*), and smoky brown cockroach (*Periplaneta fuliginosa*) have been reported to cause allergic asthma morbidity worldwide (2). Cockroach allergy is predominantly caused by *Blattella* and *Periplaneta* in temperate and (sub)tropical areas and is a global health problem due to the increasing infestation of cockroaches in human housing environments (3). Cockroach allergens are present in saliva, fecal particles, spermatophores, shredded skin, and desiccated remains of insect bodies (2). In studies including children and adults, presented to hospital, the prevalence of cockroach allergy ranges from 17 to 41% in the United States, and 60–80% of inner-city children with asthma were sensitized to cockroach. It has been suggested that exposure to cockroach allergens appears to have a greater effect on asthma morbidity than dust mite or pet allergens, in particular among inner-city children (2).

Allergic sensitization to cockroach is frequently investigated by skin testing using crude extracts and by the measurement of specific IgE to cockroach allergens (2). However, the usage of allergen extracts possesses some limitations. Commercial or self-prepared allergen extracts at most are standardized based on in-house assays, causing the lack of comparability between different manufacturers. Moreover, the lack of immune-dominant allergen(s) and the complex patterns of IgE responses to cockroach extracts have made it difficult to produce standardized cockroach allergen extracts (4). Therefore, one approach to overcome these drawbacks is the utilization of purified allergens for component-resolved diagnostics (CRD).

Whereas, allergic sensitization to *B. germanica* allergens has been investigated in detail, data referring to *Periplaneta* allergens are less available. However, the tested cockroach species frequently is not indicated in the respective reports. Of note, species-specific sensitization to *Periplaneta* and *Blattella* has been reported, and in one study, only 68% of *Blattella*-reactive patients showed sensitization to *Periplaneta* (5). *Vice versa*, in China, 25.7% of allergic patients were sensitized to *Periplaneta* allergens, whereas only 18.7% were sensitized to *Blattella* allergens (6), indicating allergenic differences between these cockroach species or different diagnostic sensitivity of the extracts used. Although homologous allergens have been described for *Periplaneta* and *Blattella*, the presence of different allergenic components between the species needs to be considered. So far, among 13 suggested *Periplaneta* allergens, 11 have been officially recognized by the WHO/IUIS Allergen Nomenclature Sub-Committee (7, 8). Per a 1 (25–45 kDa, midgut microvilli-like protein) (9–11), Per a 2 (42 kDa, aspartic protease-like protein) (12), Per a 3 (46–79 kDa, homologue of arylphorin and insect hemocyanin) (13, 14), Per a 5 (23 kDa, glutathione S-transferase) (15–17), Per a 6 (18 kDa, troponin C) (18), Per a 7 (33 kDa, tropomyosin) (19, 20), Per a 9 (43 kDa, arginine kinase) (21, 22), Per a 10 (28 kDa, serine protease) (23), Per a 11 (55 kDa, α-amylase) (24), Per a 12 (45 kDa, chitinase) (24), and Per a 13 (36 kDa, glyceraldehyde-3-phosphate dehydrogenase) (24). Although lipocalin (Acc.No. AY792948) (25, 26) and myosin light chain (Acc.No. JQ279816, unpublished) have been suggested as potential *Periplaneta* allergens and have been proposed as Per a 4 and Per a 8, they are less well-characterized.

So far, studies addressing the sensitizing capacity of *Periplaneta* allergens were mainly performed with selected allergens rather than by CRD using the panel of *Periplaneta* allergens. Of note, data on the prevalence of IgE binding to target allergens are highly variable between different studies (7). An ELISA-based CRD study conducted in Taiwan, enrolling *Blattella*-sensitized patients with persistent asthma and including recombinant Per a 1 through Per a 7 and Per a 9, revealed a reactivity with Per a 2 and Per a 9 to be associated with severe asthma and allergic rhinitis, respectively (12). Taken together, the reports on the contribution of individual *Periplaneta* allergens in cockroach allergy are not consistent. It is worth noting that study results on the clinical significance of individual *Periplaneta* allergens may vary substantially depending on the quality of purified allergens used for antibody detection, patient inclusion criteria, co-exposure to cross-reactive allergens from other sources, and the geographical area where the study is conducted.

The present study aimed to investigate the molecular sensitization profile in a substantial number of cockroach-sensitized patients recruited in four different countries, all with perennial respiratory indoor allergy. For the purpose of CRD, a set of nine recombinant *Periplaneta* allergens were produced and characterized by uniform methods.

## Materials and Methods

### Patients and Patient's Sera

For CRD, patients (*n* = 117) reporting perennial respiratory indoor allergy with confirmed IgE sensitization to *P. americana* and/or *B. germanica* were included in the study. Patients' sensitization to cockroach was monitored by IgE-ELISA (India, *n* = 35) and IgE-immunoblotting (Iran, *n* = 30) using the self-prepared *Periplaneta* extract, by SPT using *Blattella* extract (ALK, Round Rock, TX, United States) (Venezuela, *n* = 25), and *Blattella* extract ImmunoCAP (i6, Thermo Fisher Scientific, Uppsala, Sweden) (Korea, *n* = 27), respectively. Screening of *Periplaneta* allergen expression and purification was done using sera from cockroach-allergic patients (Germany, *n* = 1; Plasmalab, Everett, WA, United States, *n* = 6). Ethical approval was obtained from IHEC (no. CLP 0019, CSIR-IGIB) and Isfahan University of Medical Sciences and Health Services (no. 295264).

### Preparation of *P. americana* Extract

*Periplaneta americana* extract was produced as described in the Supplementary Material. In brief, proteins were extracted from lyophilized powder of defatted whole-body *Periplaneta* cockroaches using phosphate-buffered saline (PBS), and the protein fractions were gained after subsequent centrifugation and filtration steps.

### Generation and Physicochemical Characterization of Recombinant *Periplaneta* Allergens

Detailed steps for the preparation of recombinant Per a allergens are depicted in the Supplementary Material. For cDNA cloning, published GenBank amino acid (AA) sequences were used as template, and signal peptides were excluded from the sequence. Five out of the nine proteins were produced as non-tagged proteins. Per a 2 and Per a 3 contain a C-terminal His_tag_, whereas Per a 5 and Per a 10 comprise a N-terminal His_tag_ for purification. All proteins were expressed in *Escherichia coli* and purified by multiple chromatographic steps. Final protein concentration and purity was checked by bicinchoninic acid (BCA) (Sigma-Aldrich, United States), amino acid analysis (AAA), and sodium dodecyl sulfate–polyacrylamide gel electrophoresis (SDS–PAGE). Additionally, mass spectrometry (MS) (intact masses and tryptic digestion) analysis was performed to confirm the identity of recombinant proteins. To determine the structural integrity and the aggregation status of the proteins, circular dichroism (CD), and dynamic light scattering (DLS) were carried out. Recombinant expression of individual allergens and analytical methods are described in the Supplementary Material.

### IgE Immunoblot and CRD

Immunoblotting was performed to (1) demonstrate IgE sensitization of Iranian patients recruited by clinical history (not shown) and (2) to assess the IgE sensitization pattern for selected patients lacking detectable IgE binding to purified *Periplaneta* allergens. Briefly, *P. americana* extract (50 μg/cm) was applied to SDS–PAGE, transferred to nitrocellulose membrane (0.2 μm, Amersham Protean, GE Healthcare, Freiburg, Germany) by semi-dry blotting, and visualized by Ponceau S staining (Sigma-Aldrich, Munich, Germany). After blocking with 0.3% Tween-20 in Tris-buffered saline (TBS, 50 mM Tris, 150 mM NaCl, pH 7.4), the membrane was incubated overnight with patient's serum (500 μl/strip, diluted 1:10 in TBS, 0.05% Tween-20, 0.1% BSA), and bound IgE was detected as described elsewhere (27).

Specific IgE values to *Periplaneta* (i206) and *Blattella* extract (i6) were determined by ImmunoCAP tests (Thermo Fisher Scientific) and to single recombinant *Periplaneta* allergens by experimental ImmunoCAP tests as previously described (28). Assay background of each test was assessed and cutoff level for positivity adapted as required (0.35 kU_A_/L for Per a 2, Per a 5, and Per a 10 and 0.10 kU_A_/L for all other allergens).

## Results

### Generation of Recombinant *Periplaneta* Allergens

**Figure 1:**
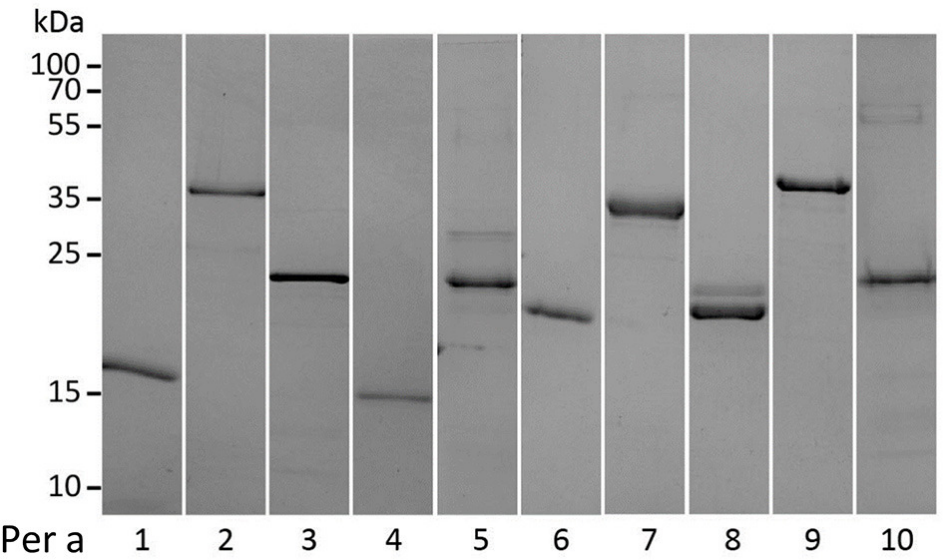
SDS–PAGE and Coomassie staining of recombinant *Periplaneta* allergens, Per a 1 to Per a 10 (1-10), under reducing conditions.

All recombinant *Periplaneta* allergens were expressed in *E. coli* as non-fusion as well as N- or C-terminal His_tag_ proteins. After multistep chromatography, purified proteins were characterized by uniform physicochemical methods as indicated in Supplementary Table 1. Purity and apparent molecular weight of all proteins were assessed by SDS–PAGE performed under reducing conditions (Figure 1).

The identity of all nine recombinant *Periplaneta* proteins included in the study was confirmed by MS analysis, by either intact mass analysis or in-solution/in-gel digestion and AA analysis. The identity of Per a 6 could not be confirmed, and it was therefore excluded from the study. Of note, the MS analysis revealed that a high proportion of Per a 8 had a truncation at the N-terminus of the protein, with only 10% being present as the full-length protein (Supplementary Table 1). The truncation observed for Per a 8 did not affect the secondary structure of the protein. CD spectroscopy confirmed that most of the allergens show structural integrity or were at least partially folded (Supplementary Table 1). Analysis by DLS revealed that some recombinant allergens tended to partially aggregate; however Per a 4, Per a 8, and Per a 9 were determined as mostly monomeric proteins.

### IgE Sensitization to *Periplaneta* and *Blattella* Allergens

**Figure 2:**
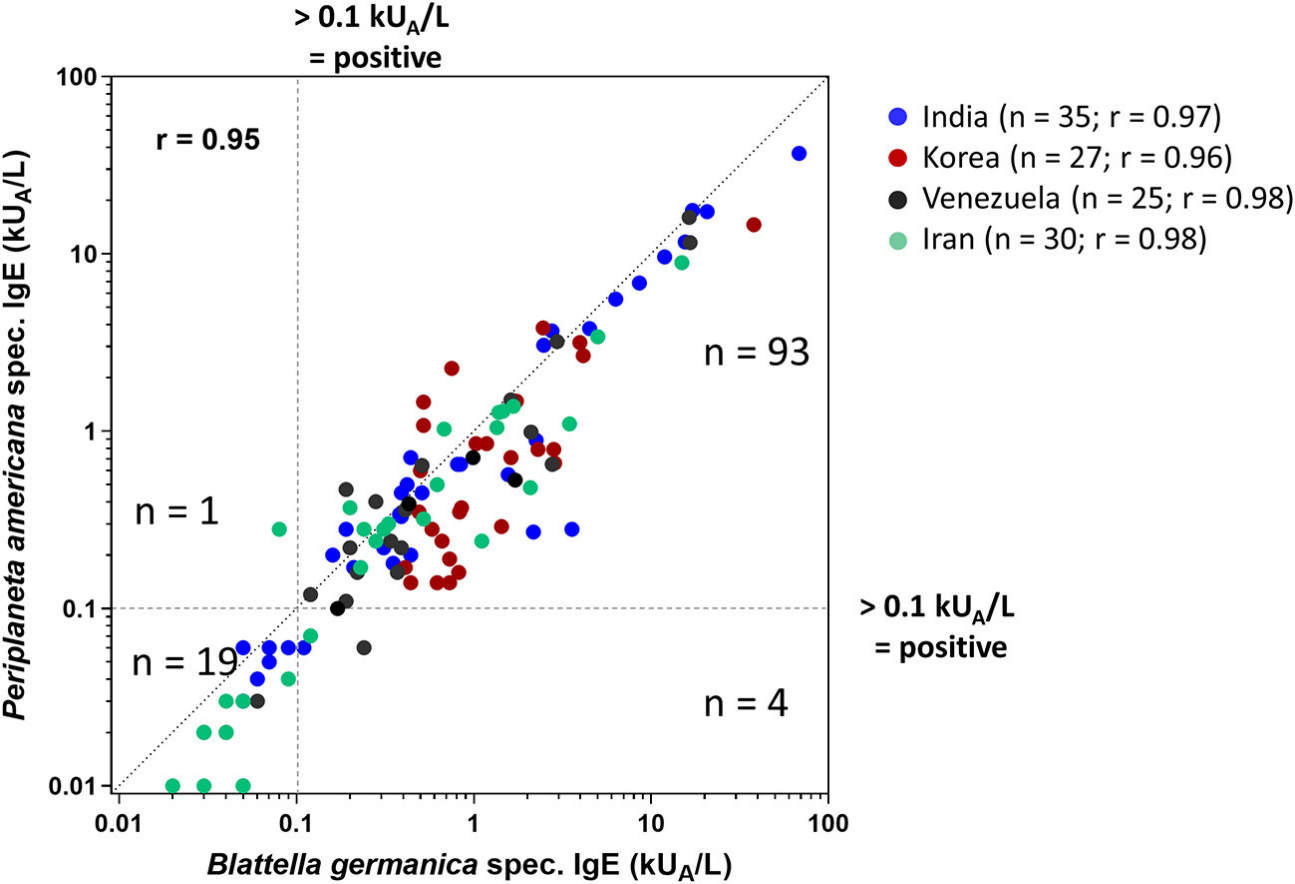
Comparison of *Periplaneta* (i206)- and *Blattella* (i6)-specific IgE values in 117 patients (India, *n* = 35; Korea, *n* = 27; Venezuela, *n* = 25; Iran, *n* = 30) reporting cockroach allergy by ImmunoCAP testing (cutoff 0.1 kU_A_/L); *r*-values are indicated.

*Periplaneta*- and *Blattella-*specific IgE was analyzed by ImmunoCAP testing in all 117 serum samples (Figure 2), showing an overall interspecies correlation of *r* = 0.95 and *r* = 0.96–0.98 for individual patient collectives. *In vitro* IgE reactivity to *Blattella* and *Periplaneta* was confirmed in 83% (97/117) and 81% (95/117) of the patients, respectively. Four subjects were sensitized to *Blattella* but not to *Periplaneta*, and one was sensitized to *Periplaneta* but not to *Blattella*. In total, 98 of 117 patients reacted to one or both of the cockroach species (Figure 2). Although patients were preselected by sensitization to cockroach by ELISA, SPT, and immunoblotting, 19/117 sera (comprising 6/35 from India, 3/25 from Venezuela, 10/30 from Iran) were tested negative to both species by ImmunoCAP.

### Component-resolved Diagnosis of *Periplaneta* Allergy by ImmunoCAP Testing

**Figure 3:**
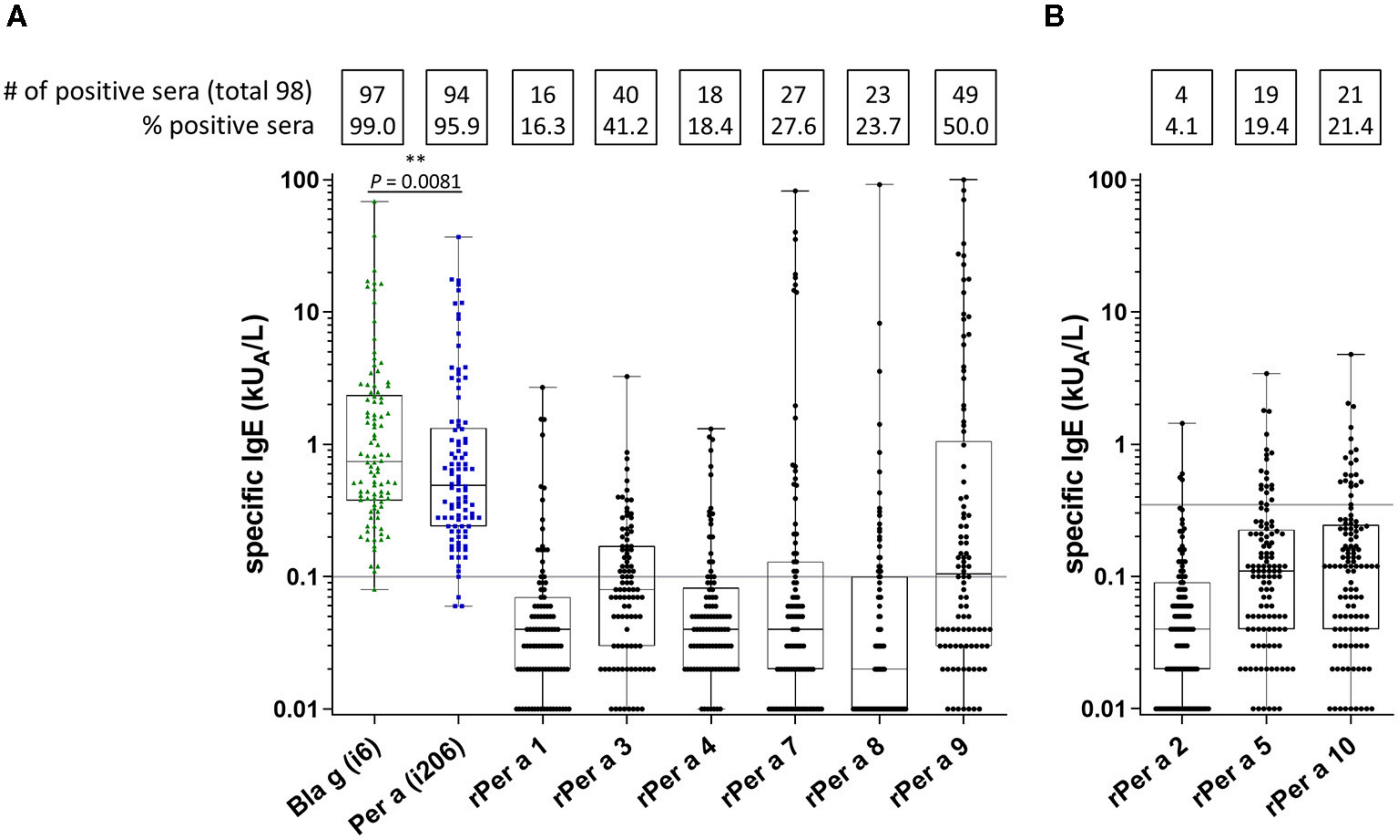
CRD of cockroach allergy by experimental ImmunoCAP testing including cockroach-allergic patients preselected by positive *Periplaneta-* or *Blattella*-specific ImmunoCAP values (*n* = 98). Specific IgE values for *Periplaneta* and *Blattella* extracts and Per a 1, Per a 3, Per a 4, Per a 7, Per a 8, and Per a 9 (cutoff 0.1 kU_A_/L) **(A)**, as well as for Per a 2, Per a 5, and Per a 10 (cutoff 0.35 kU_A_/L) **(B)** are depicted. (paired *t*-test).

In total, nine recombinant *Periplaneta* allergens (Per a 1 to Per a 10, except Per a 6) were included for CRD by experimental ImmunoCAP testing. Since the preparation of Per a 2, Per a 5, and Per a 10 displayed a tendency of unspecific IgE binding, the threshold for test positivity was set to 0.35 kU_A_/L for these allergens (Figure 3B). Other *Periplaneta* allergens were evaluated with a cutoff of 0.1 kU_A_/L (Figure 3A). Although no major allergen could be identified, Per a 3 and Per a 9 appeared as the most important in this study. The frequencies of sensitization to the different allergens in the entire study population were as follows: Per a 1: 16% (16/98), Per a 3: 41% (40/98), Per a 4: 18% (18/98), Per a 7: 28% (27/98), Per a 8: 24% (23/98), and Per a 9: 50% (49/98) (Figure 3A). Per a 4 and Per a 8 were characterized as minor allergens in comparison with other *Periplaneta* allergens. The frequency of IgE recognition of Per a 2, Per a 5, and Per a 10 was 4, 19, and 21%, respectively (Figure 3B).

**Figure 4:**
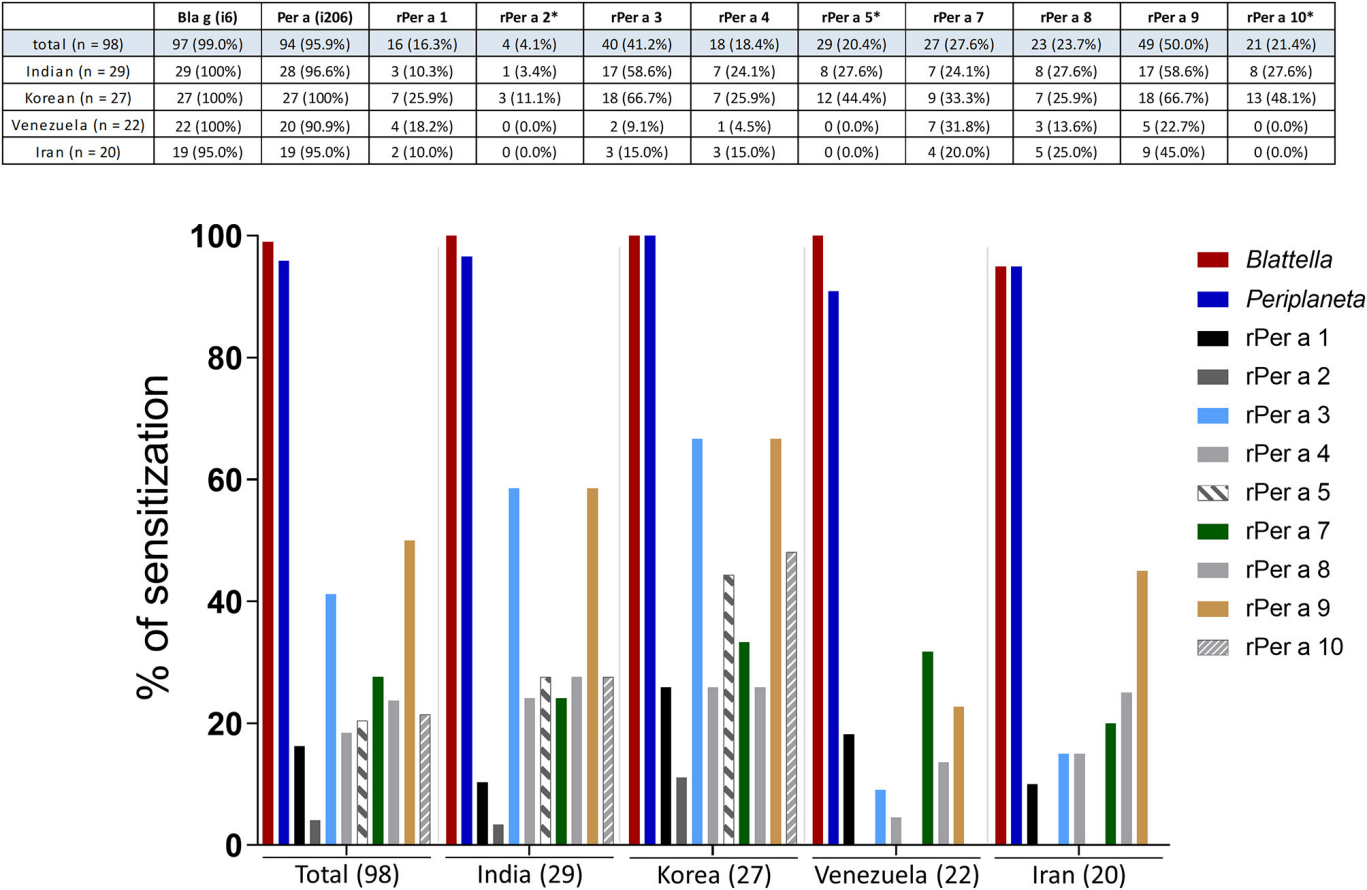
Comparison of IgE antibody levels to *Periplaneta* (i206), *Blattella* (i6), and purified recombinant *Periplaneta* allergens in respective patient collectives from India (*n* = 29), Korea (*n* = 27), Venezuela (*n* = 22), and Iran (*n* = 20). Prevalence (%) of IgE sensitization to Per a 1, Per a 3, Per a 4, Per a 7, Per a 8, and Per a 9 (cutoff of 0.1 kU_A_/L) and Per a 2*, Per a 5*, and Per a 10* (cutoff of 0.35 kU_A_/L) are shown.

The sensitization pattern within each patient group was diverse and not comparable between the different patient collectives (Figure 4). Analysis of individual patient groups from different geographical areas revealed the highest prevalence of IgE reactivity to Per a 3 (59 and 67%) and Per a 9 (57 and 67%) in Indian and Korean patients. Per a 9 was the most prominent (45%) allergen also among the Iranian patients, whereas patients from Venezuela reacted predominantly to Per a 7 (31%) (Figure 4). Per a 4 and Per a 8 were identified as minor allergens in patients from almost all investigated geographies. However, the prevalence of sensitization to these components was the lowest among subjects from Venezuela. Patients from India and Korea showed low frequencies of IgE reactivity to Per a 2, Per a 5, and Per a 10 (Figure 4) (India: 3% to Per a 2, 28% each to Per a 5 and 10; Korea: 11% to Per a 2, 44% to Per a 5 and 48% to Per a 10). Notably, none of the Venezuelan or Iranian patients were sensitized to any of these three proteins (Figure 4).

Of the 95 *Periplaneta* ImmunoCAP-positive patients, 28 (29%) did not react with any of the purified allergens tested (Supplementary Table 2). This discrepancy was most prominent in serum samples from Iran 9/19 (47%), but less frequent in samples from India 6/28 (21%), Korea 7/27 (26%), and Venezuela 6/21 (29%). It is tempting to speculate that these patients are sensitized to other *Periplaneta* proteins or isoforms not reflected by the present CRD panel. In contrast, only 4% (5/117) of the patients showed IgE sensitization (with IgE levels close above the cutoff) to one or more of the purified allergen(s) despite a negative test result with *Blattella* and *Periplaneta* ImmunoCAP.

### IgE Immune Reactivity of Periplaneta Extract

**Figure 5:**
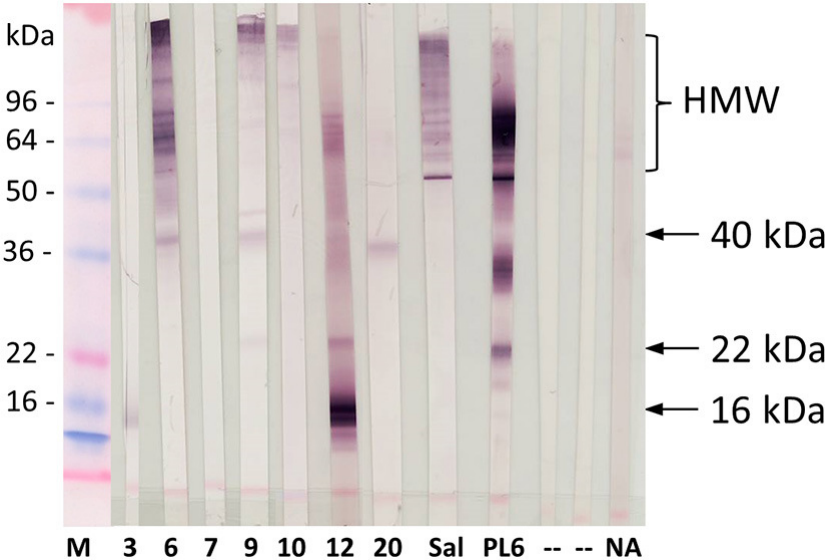
IgE-reactivity pattern of cockroach-reactive patients from Venezuela (*n* = 7, VR3, VR6, VR7, VR9, VR10, VR12, VR20) showing negative ImmunoCAP results for recombinant *Periplaneta* allergens, Germany (Sal), and pooled sera from cockroach-sensitized patients (PL6) with *Periplaneta* extract. M, marker; P, Ponceau S, staining; NA, non-atopic control; “-”, secondary antibody control. Arrows indicate the putative yet not identified allergens.

Sera of cockroach-sensitized patients (*n* = 7) from Venezuela either lacking any IgE reactivity (VR03, VR06, VR10, VR20) or showing very low IgE values of 0.12 kU_A_/L (VR07, VR12) to purified allergens were further analyzed by immunoblotting using *Periplaneta* extract (Figure 5). Two sera (Sal and PL6) were applied as positive controls. IgE binding to putative novel and yet not identified *Periplaneta* proteins was demonstrated for six out of seven sera, showing IgE binding to distinct high molecular weight (HMW) proteins and proteins with an apparent molecular weight of 16 kDa (VR3, VR12), 22 kDa (VR12), and 40 kDa (VR6, VR9, and VR20) not verified so far.

However, the accumulation of both proteins, lipocalin and myosin light chain, in *Periplaneta* extract was confirmed by MS/MS analysis. Peptides derived from both allergens showed a sequence coverage of 15 and 29% (for Per a 4) and 34 and 59% (for Per a 8), in two extracts analyzed independently (Supplementary Figure 3).

## Discussion

Cockroaches are an important source of indoor aeroallergens. However, allergens from American cockroach (*P. americana*) are less investigated in comparison with German cockroach (*B. germanica*) allergens, and reports on the involvement of individual *Periplaneta* allergens in allergy related morbidity are inconsistent. Notably, clinically relevant sensitization to *Blattella* across Europe was reported in the GA^2^LEN skin test study (29). In addition, sensitization to different cockroach species has been reported in South Italy (30), demonstrating positive RAST results to *Periplaneta* in four of 15 cockroaches (mixed species) SPT-positive patients (31). However, in Italian children, sensitization was substantially higher to *Periplaneta* than to *Blattella* (32). So far, the molecular sensitization profile to *Periplaneta* allergens was not addressed in these studies, except one study describing reactivity to Per a 7 (tropomyosin) in 41% of *Periplaneta*-sensitized indoor allergic patients from Marseille (19). All these studies implicate that cockroach allergen exposure and sensitization are a global health problem that will likely increase along with global warming. This prompted us to investigate the involvement of *Periplaneta* allergens in the sensitization profile to cockroach.

In the present study, using different patient collectives, a substantial panel of recombinant *Periplaneta* allergens, including yet less well-characterized allergens Per a 4 and Per a 8, was utilized for CRD employing a uniform analytical methodology. The identity of all purified *Periplaneta* allergens used in the study was confirmed by MS. We attempted to produce Per a 6 but were unable to confirm its identity. Three known *Periplaneta* allergens Per a 11 to Per a 13 were not included in the study since they were not yet been identified at the time the study was initiated.

A total of 117 patients from four countries with subtropical or tropical climates, with perennial respiratory indoor allergy, and evidence of IgE sensitization to cockroach (evaluated by *in vitro* and *in vivo* methods), were included in the study. Of note, the limitation of the study is the lack of consistent inclusion criteria in terms of clinical cross-reactivity (co-sensitization with cross-reactive mite and insect allergens), and the application of different IgE detection methods in different cohorts, which can lead to a bias of the preselected patients. Cockroach-specific IgE was re-evaluated and confirmed in 81% and 83% of all sera by commercial ImmunoCAP testing, using *Periplaneta* or *Blattella* extracts, respectively. Whereas, sera from Korean patients were preselected by positive ImmunoCAP results, IgE sensitization of patients from Venezuela, India, and Iran was assessed by either skin testing, ELISA, or immunoblotting, showing a correlation with *Periplaneta*-positive ImmunoCAP testing between 63 and 80%. Discrepancy between results from ImmunoCAP and other test systems might be due to various applications of non-standardized and self-prepared extracts, as well as the different diagnostic sensitivity of each test system.

ImmunoCAP testing showed an almost similar diagnostic sensitivity for *Blattella* and *Periplaneta* extract and levels of IgE to the two cockroach species were strongly correlated (*r* = 0.95). Only one patient with low *Periplaneta*-specific IgE but negative *Blattella*-specific IgE values was detected. *Vice versa*, four patients with reactivity to *Blattella*, but without sensitization to *Periplaneta*, were identified. Finally, the diagnostic sensitivity using recombinant allergens was increased for five patients who did not react with any of the two extracts. The overall concordant results are likely explained by the presence of conserved and cross-reactive allergens in *Periplaneta* and *Blattella*, consistent with the official recognition of allergens from at least seven groups identified in both species. However, only partial IgE cross-reactivity between members of homologous groups has been reported (2). Slight variability of IgE-binding capacity between the species might be due to distinct expression levels of certain allergens in cockroaches, a variable presence of allergens in the applied extracts, or the abundance of species-specific allergens. In line with this, group 4 and 8 allergens were already denominated according to the IUIS allergen nomenclature database for *Blattella* but not for *Periplaneta*, whereas group 10 and 13 allergens have so far not been identified in *Blattella*. In the present study, we produced recombinant *Periplaneta* proteins homologous to Bla g 4 and Bla g 8 and included them in the panel of allergens used for CRD.

Component-resolved diagnostics was performed with patients (*n* = 98) reporting perennial respiratory indoor allergies and IgE sensitization to *Blattella* or *Periplaneta*, confirmed by ImmunoCAP testing as a uniform methodology across the entire population. Results showed only 68% (67/98) of ImmunoCAP-positive patients to react with any of the nine recombinant *Periplaneta* allergens tested. However, a study addressing CRD of *Blattella* allergy suggested a panel of only four allergens (Bla g 1, Bla g 2, Bla g 4, and Bla g 5) to be sufficient to identify 95% of cockroach-allergic patients in the United States (33). In another study, all patients with airway allergy and positive *Blattella* ImmunoCAP values were reported to react with at least one of Per a 1 to 7 and Per a 9, tested by ELISA (12). In contrast, our study is in agreement with other reports demonstrating that a set of several recombinant *Blattella* allergens fall short of reflecting the IgE reactivity of corresponding natural *Blattella* allergen extract (34, 35). By applying a panel of allergens, only 49% and 62% of cockroach-allergic patients were tested positive either by skin testing (35) or by *in vitro* assays (34), respectively. Both studies are in accordance with our present results showing a higher diagnostic sensitivity of the natural extract in comparison with the applied recombinant allergens. The higher diagnostic value of cockroach extract in comparison with the panel of purified allergens can be explained by missing allergens which need to be included in CRD, structural modification of recombinant allergens affecting the IgE reactivity (e.g., mediated by posttranslational modifications, or partial aggregation of recombinants). Furthermore, the presence of IgE-reactive natural variants/isoforms that are not reflected by the selected recombinant allergens could play a role in the lower diagnostic sensitivity of the CRD.

The present study demonstrated a heterogeneous pattern of IgE sensitization to *Periplaneta* allergens in different patient collectives. The IgE-reactivity profiles could be in part due to the exposure and IgE responses to abundant cross-reactive homologous allergens. For all *Periplaneta* allergens except Per a 1, homologous groups and potential cross-reactive allergens have been described among invertebrates other than cockroaches (1). In agreement with a previous notion by Pomes et al. (1), we did not observe any dominant and thus major *Periplaneta* allergen considering the whole study collective. Importantly, the analysis of the individual geographical different cohort revealed Per a 3 and Per a 9 as major allergens (with IgE prevalence ≥50% in the respective patient group) in India and Korea. This, together with the occurrence of extract-positive subjects without IgE to any of the components included in this study, indicates the need to identify and evaluate additional *Periplaneta* allergens in each cohort.

In the present study, lipocalin and myosin light chain were classified for the first time as minor *Periplaneta* allergens in all investigated cohorts with an overall frequency of IgE sensitization of 18 and 24%, respectively. Both proteins were accepted by the WHO/IUIS Allergen Nomenclature Sub-Committee as Per a 4 and Per a 8. Sensitization to recombinant Per a 4 was most prevalent in patients from Korea (26%), and sensitization to recombinant Per a 8 was most prevalent in Indian patients (28%). Whether the IgE-binding capacity of recombinant allergens is reflected by the natural counterparts remains open. However, our study provided evidence that the prominence of allergens is different in the respective patient collective. The results are in agreement with a limited number of studies describing molecular sensitization profiles to *Periplaneta* allergens and showing a heterogeneous clinical significance of single allergens. In line with this, the prevalence of IgE sensitization of 5–100% for Per a 1, 63% for Per a 2, 26–95% for Per a 3, 30–70% for Per a 5 (16), 13–54% for Per a 7, 80–100% for Per a 9, 82% for Per a 10, 83% for Per a 11, and 64% for Per a 12 has been reported (7). A retrospective study of 118 cockroach-sensitized subjects from inner-city environments in the United States revealed that only 13% were sensitized to Per a 7 (34). In contrast, Per a 7 was found to be the dominant allergen among 55 cockroach-allergic patients in Brazil, with a prevalence of IgE reactivity of 42% (35).

Our data suggested the involvement of yet unidentified *Periplaneta* allergens (including isoforms and variants) and/or a potential important role of Per a 6, Per a 11 to Per a 13, which were not tested in the current study. Moreover, 2D-immunoblotting and subsequent MS analysis using pooled sera, not reactive to any of the tested recombinant Per a allergens, revealed IgE binding to 12- and 16-kDa proteins, in addition to a 40-kDa band which might correspond to Per a 11 to Per a 13 (data not shown). In addition, the role of protein glycosylation in the immunogenicity of cockroach allergy (2) needs to be considered. Insects express immunogenic core α-1,3 carbohydrate structures with additional −1,6 fucosylation (36), and glycan modification of *Blattella* allergens has been described (37, 38). Although reports on cross-reactive carbohydrate determinants (CCD) of cockroach allergens are limited, and routine diagnosis lacks insect-specific glycan moieties, it is tempting to speculate that a substantial number of cockroach-sensitized patients are reactive to glycan structures which in part may explain our observed gap in diagnostic sensitivity between natural cockroach extracts and a panel of recombinant, non-glycosylated allergens.

In summary, we show that levels of IgE antibodies to *Periplaneta* and *Blattella* are strongly correlating and that natural *Periplaneta* extract displays higher diagnostic sensitivity than a panel of nine recombinant *Periplaneta* allergens. For reasons that are elusive, patient collectives from different countries showed heterogeneous sensitization profiles. Notably, major allergens could be identified only for individual cohorts, while Per a 4 and Per a 8 were identified as minor allergens. Further improvement of *Periplaneta* CRD requires exploration of the role of isoforms, glycan determinants, and additional yet unknown allergens, which are likely classified already in other insects.

## Data Availability Statement

The datasets presented in this study can be found in online repositories. The names of the repository/repositories and accession number(s) can be found in the article/Supplementary Material.

## Ethics Statement

Ethical approval was obtained from IHEC (no. CLP 0019, CSIR-IGIB) and Isfahan university of Medical Sciences and Health services (no. 295264). Written informed consent for participation was not required for this study in accordance with the national legislation and the institutional requirements.

## Author Contributions

JS, RR, RS, SSa, FT, J-WP, and NA recruited and characterized the patients. Recombinant Per a 1, Per a 2, and Pera 3 were prepared and characterized by SE and IP. Per a 4, Per a 7, Per a 8, and Per a 9 by AW and AJ. Per a 5, Per a 6, and Per a 10 by SSh. Protein and MS analysis were performed by BS, BK, SW, GG and PB. Experimental ImmunoCAPs were established and performed by JL and FFü. NA, FFe, and SV were responsible for the study. GG, AW, and SSc coordinated the study and wrote the manuscript. All authors contributed to the article and approved the submitted version.

## Conflict of Interest

JL was employed by company Thermo Fisher Scientific. The remaining authors declare that the research was conducted in the absence of any commercial or financial relationships that could be construed as a potential conflict of interest.
