# Supplementary figures and images for: Component-Resolved Diagnosis of American Cockroach (Periplaneta americana) Allergy in Patients From Different Geographical Areas
Source: Front Allergy. 2021 Jun 29;2:691627. doi: 10.3389/falgy.2021.691627 (PMC8974670; doi:10.3389/falgy.2021.691627)

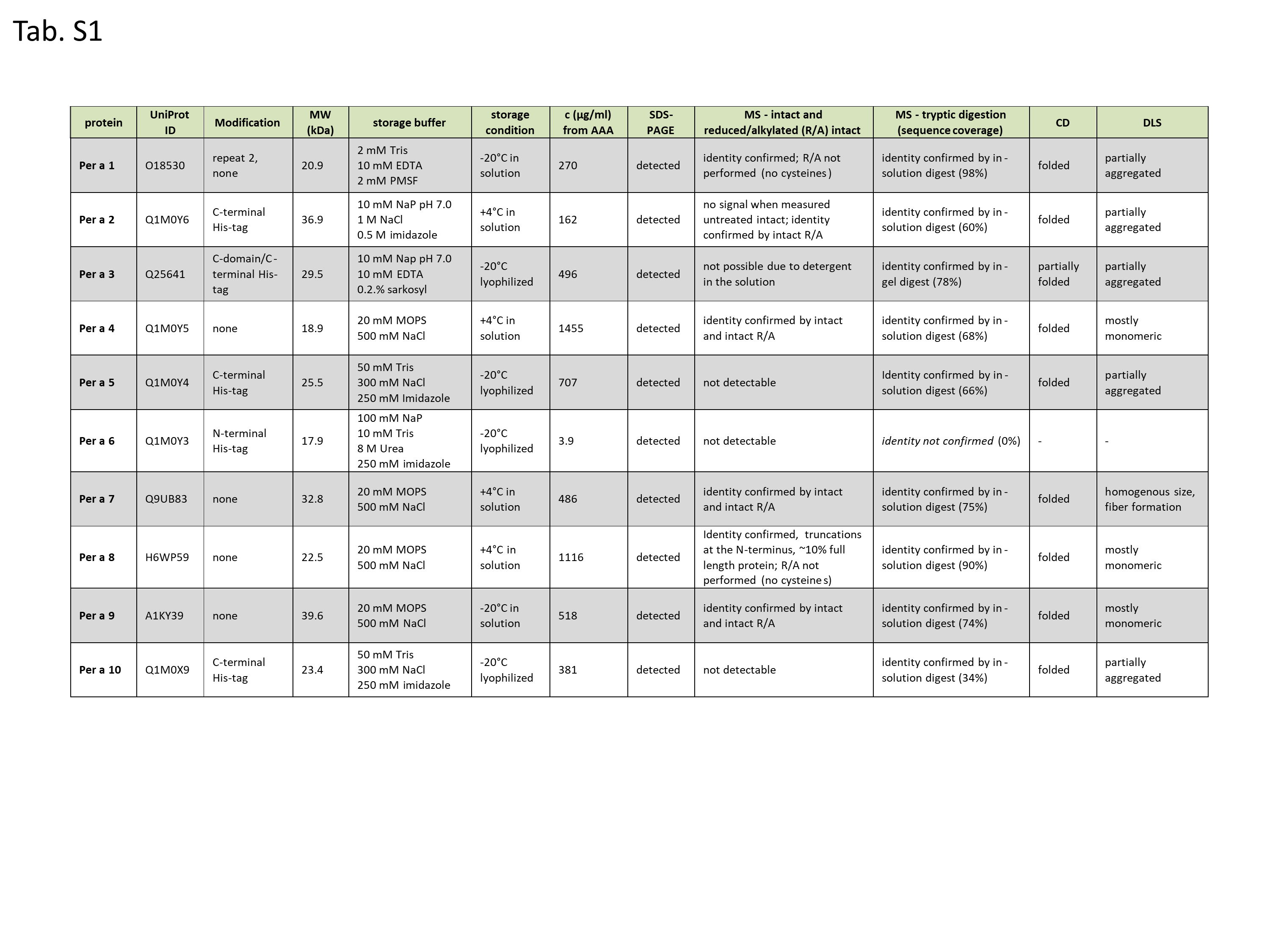

Supplement: Supplementary file 1 [file Image_1.TIF]

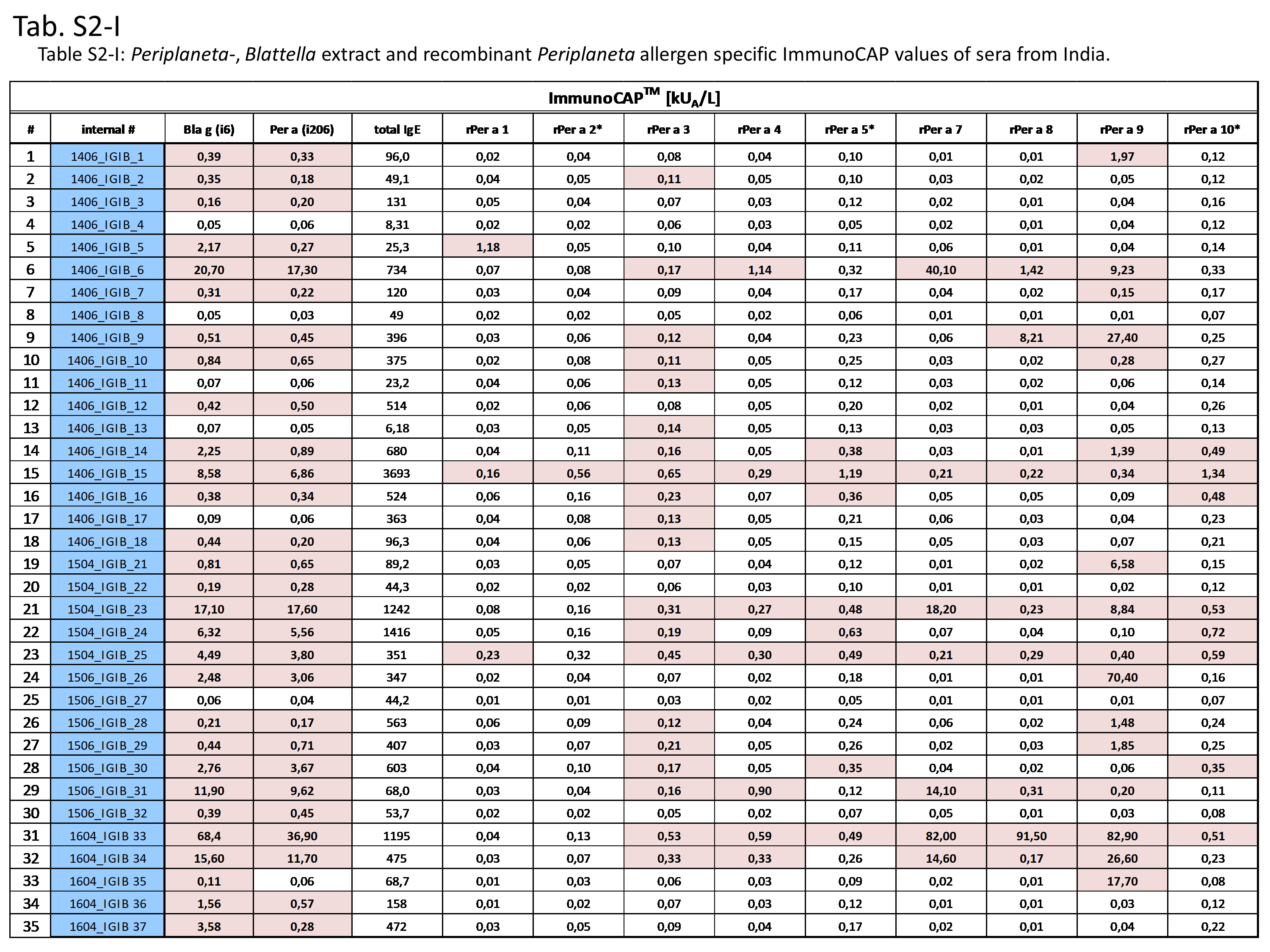

Supplement: Supplementary file 2 [file Image_2.TIF]

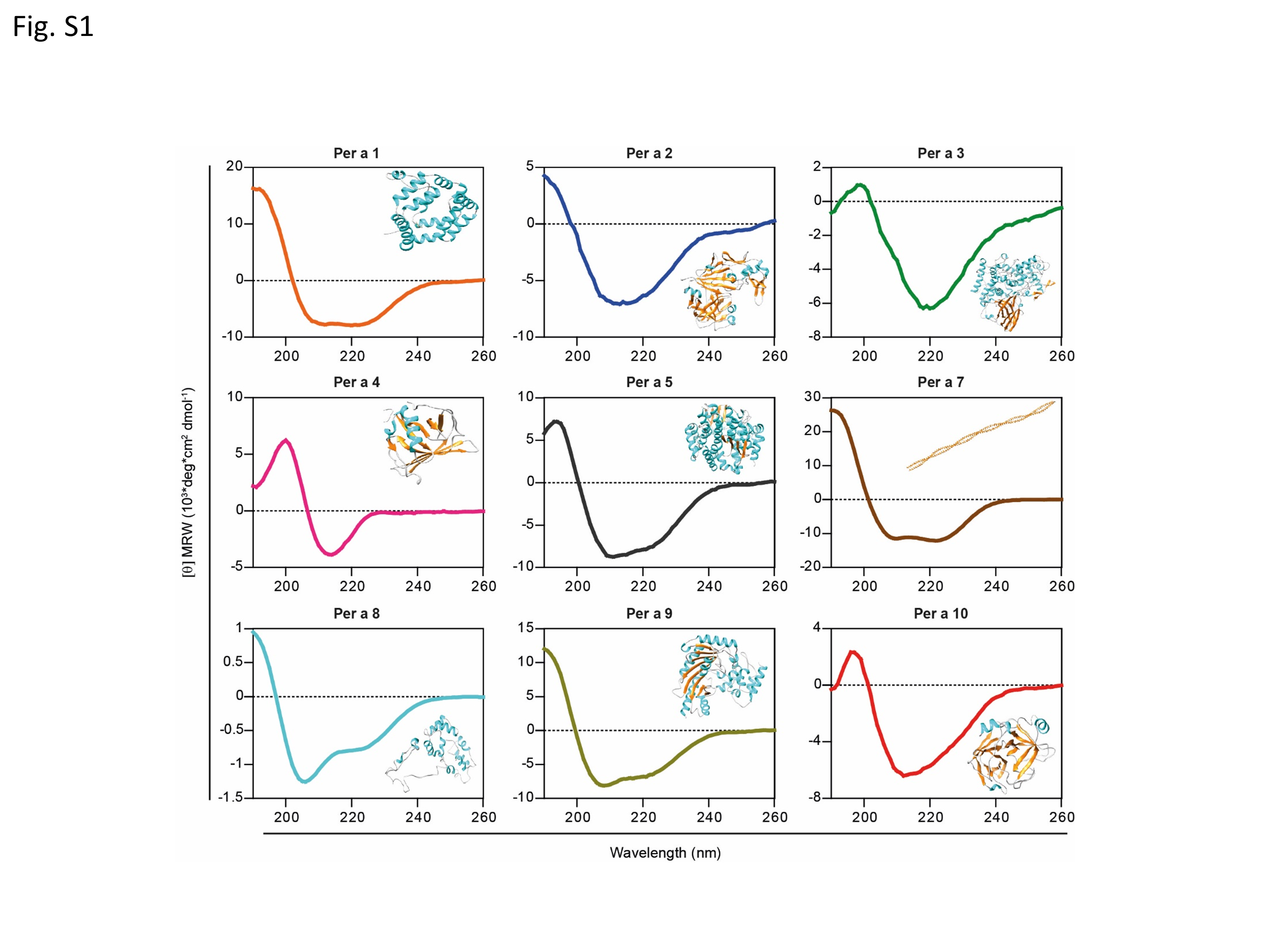

Supplement: Supplementary file 3 [file Image_3.TIF]

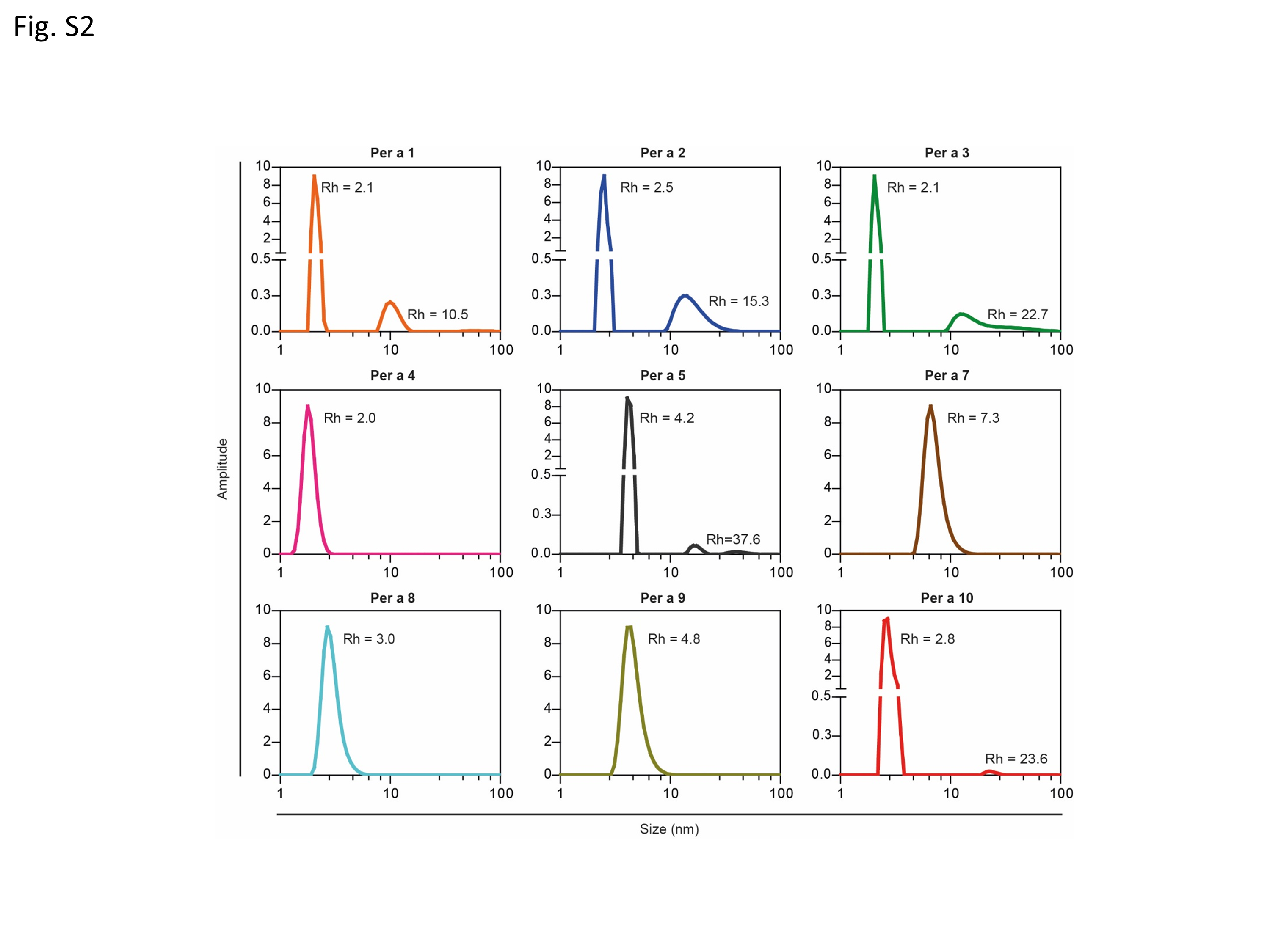

Supplement: Supplementary file 4 [file Image_4.TIF]

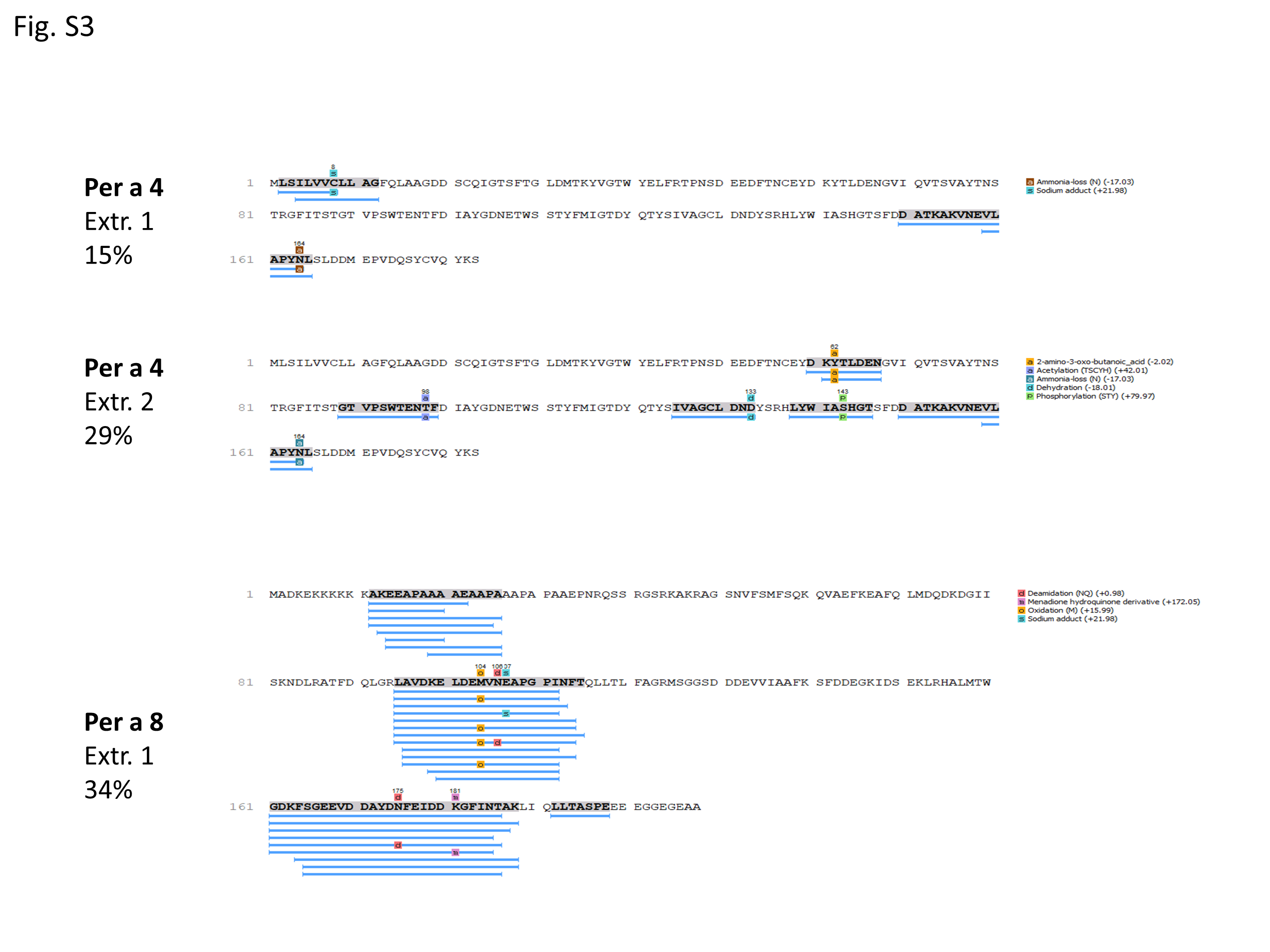

Supplement: Supplementary file 5 [file Image_5.TIF]

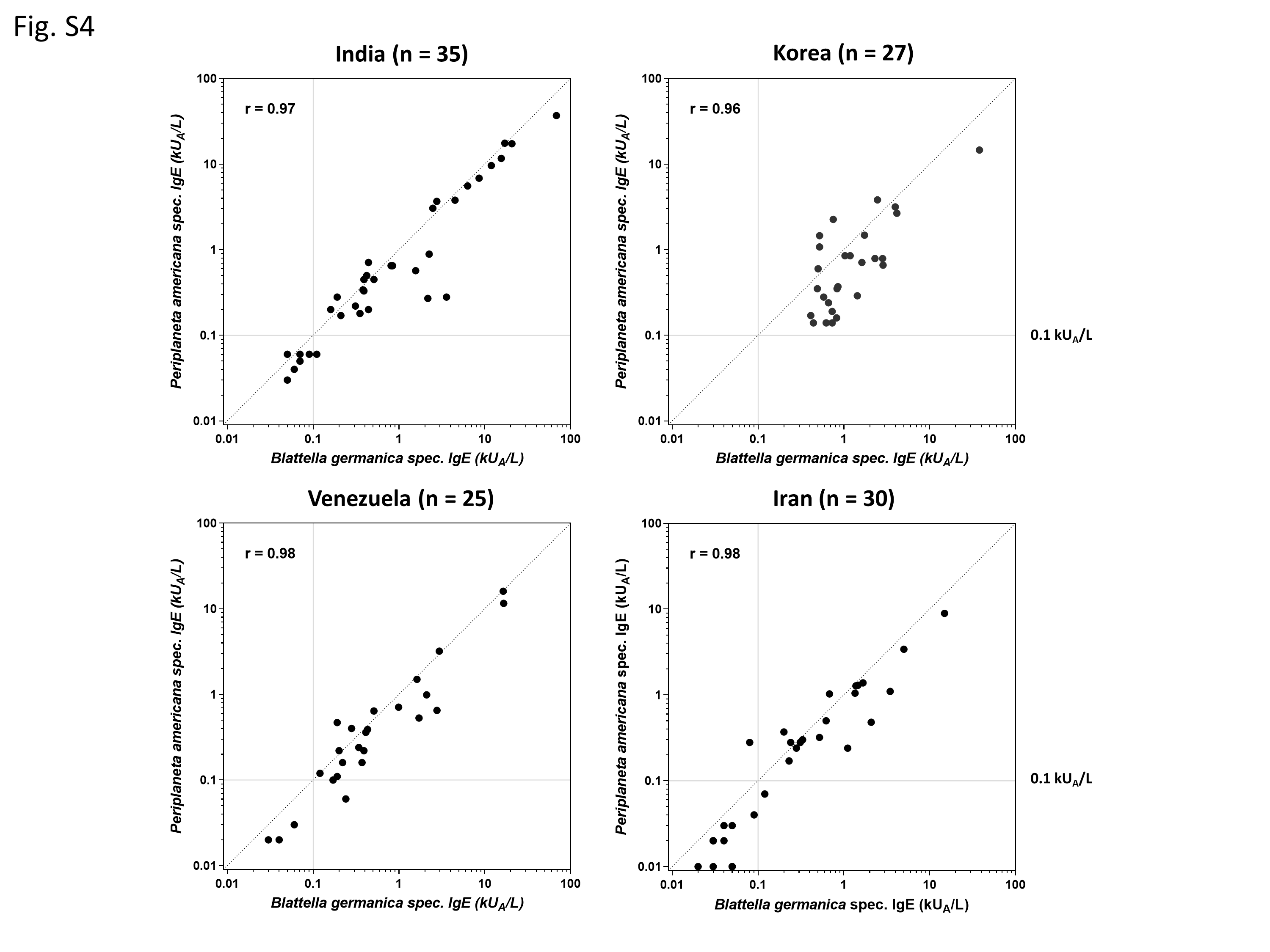

Supplement: Supplementary file 6 [file Image_6.TIF]

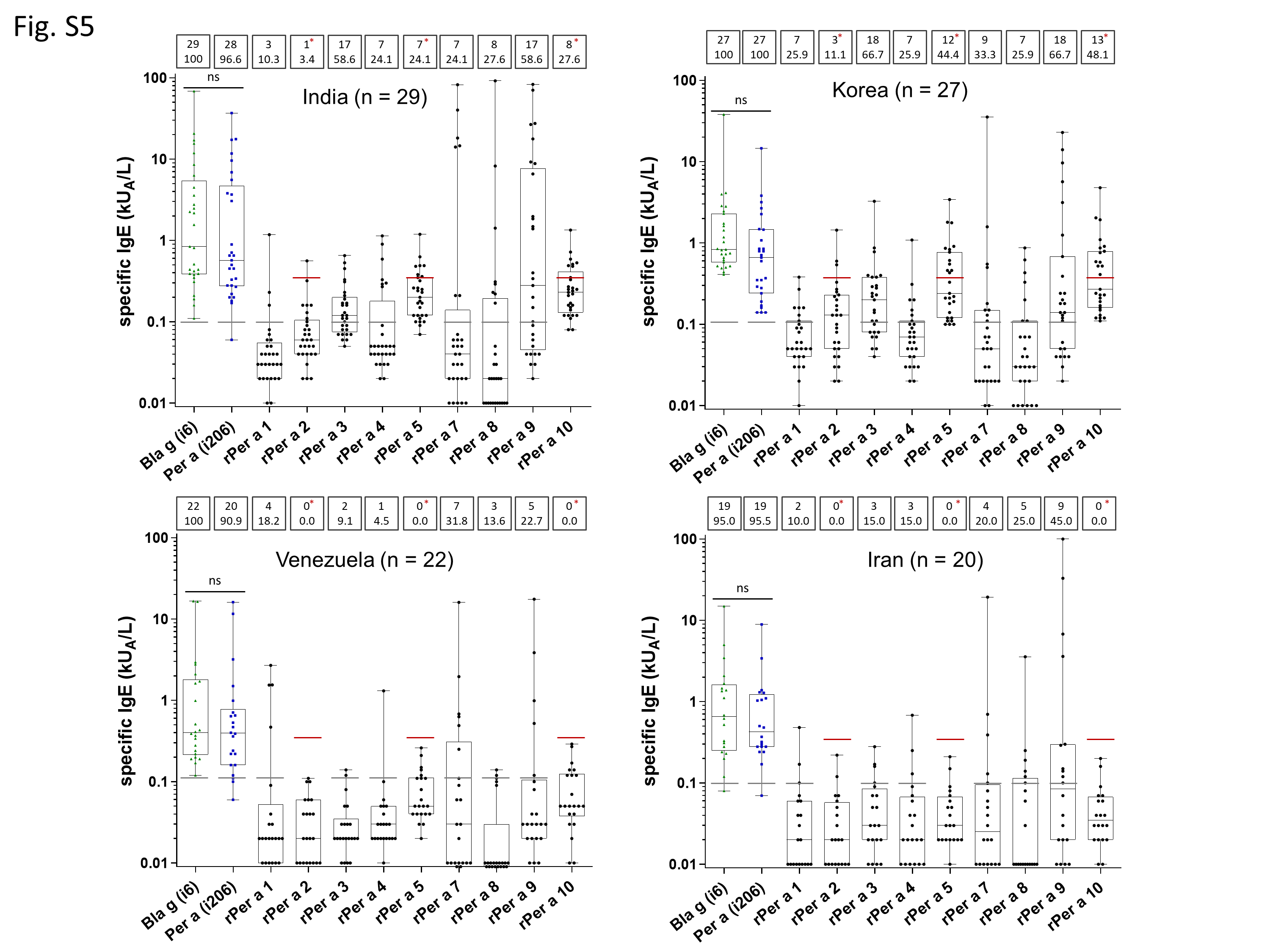

Supplement: Supplementary file 7 [file Image_7.tif]
