# Supplementary material for: Component-Resolved Diagnosis of American Cockroach (Periplaneta americana) Allergy in Patients From Different Geographical Areas
Source: Front Allergy. 2021 Jun 29;2:691627. doi: 10.3389/falgy.2021.691627 (PMC8974670; doi:10.3389/falgy.2021.691627)
Supplement: Supplementary file 8 [file Data_Sheet_1.docx]

**Component-resolved diagnosis of**

**American cockroach (*Periplaneta americana)* allergy in patients from**

**different geographical areas**

Andrea Wangorsch^1^, Annette Jamin^1^, Stephanie Eichhorn^2^, Isabel Pablos^2^, Swati Sharma^3^, Bettina Schweidler^2^, Bianca Kastner^2^, Sabrina Wildner^2^, Joachim Saloga^4^, Frank Führer^5^, Reinaldo Rafael Reyna Orozco^6^, Roya Sherkat^7^, Somayeh Sadeghi^7^, Fardis Teifoori^7^, Jung-Won Park^8^, Peter Briza^2^, Stefan Vieths^1^, Fatima Ferreira^2^, Naveen Arora^3^, Jonas Lidholm^9^, Gabriele Gadermaier^2^, Stephan Scheurer^1^

*^1^Molecular Allergology, Paul-Ehrlich-Institut, Langen (PEI), Germany*

*^2^Paris Lodron University of Salzburg (PLUS), Department of Biosciences, Salzburg, Austria*

*^3^Allergy and Immunology Section, CSIR-Institute of Genomics and Integrative Biology (IGIB), Delhi, India*

*^4^Department of Dermatology, University Medical Center of the Johannes Gutenberg-University Mainz, Mainz, Germany*

*^5^Batch Control and Allergen Analysis, Paul-Ehrlich-Institut, Langen (PEI), Germany*

*^6^Clinical Immunology Service in Hospital Militar Dr. Carlos Arvelo, Caracas, Venezuela*

*^7^Acquired Immunodeficiency Research Center, Isfahan University of Medical Sciences, Isfahan, Iran*

*^8^Yonsei University College of Medicine, Department of Internal Medicine, Seoul, Korea*

*^9^Thermo Fisher Scientific, Immunodiagnostics, Uppsala, Sweden*

**Corresponding address**

Stephan Scheurer, PhD

Molecular Allergology, Paul-Ehrlich-Institut, Paul-Ehrlich-Str. 51-59, 63225 Langen, Germany

Phone: +49 6103 775310

Email: schst@pei.de

**Keywords**

Cockroach allergy, American cockroach, *Periplaneta americana*, *Blattella germanica*, component-resolved diagnosis

**Supplementary data**

**Material and Methods**

**Preparation of *Periplaneta americana* extract**

*Periplaneta americana* allergen extract was prepared as described previously (1) (2). Briefly, freeze-dried (adult, whole body) cockroaches were powdered, defatted with diethyl ether and antigens were extracted (1:50 (w/v)) in 0.1 M phosphate buffer saline (PBS), pH 7.2, containing 5 mM EDTA (ethylenediaminetetraacetic acid; Sigma) and 1 mM phenylmethylsulfonyl fluoride (PMSF; Sigma), by continuous stirring for 8 h at 4°C. Supernatant was collected after centrifugation (10-12.000 rpm), dialysed against A.d., and lyophilized. Whole protein concentration was estimated by BCA Assay (bicinchoninic acid, Sigma). Finally the extract was subjected to ELISA or was used for immunoblotting experiments to verify IgE-binding to *Periplaneta* allergens.

To verify the accumulation of additional allergens by immunoblotting (Fig. 5) commercial ground whole body powder of *Periplaneta americana* cockroaches (Allergon, Thermo Fisher Scientific, Ängelholm, Sweden) was mixed in PBS (0.8 g/10 ml) using an Ultra-Turrax ®(IKA, Staufen, Germany) and incubated overnight (4°C) on an end over end rotator (Reax2, Heidolph-Instruments, Schwabach, Germany). The protein extract was prepared by centrifugation (12.000xg, 30’, 4°C) and filtration (5 µm, 0.8 µm and 0.45 µm). Whole protein content was determined by Roti-Nanoquant 5× Bradford reagent (Carl Roth, Karlsruhe, Germany).

**Generation of recombinant *Periplaneta* allergens**

**Per a 1**

The sequence of Per a 1 was obtained from UniProt (O18530). The predicted/putative signal peptide was not considered, and a glycine was inserted after the start-methionine for stabilization of the protein. In addition, the sequence was adapted to destroy an internal XhoI resitriction site (T576G). The gene was synthesized and cloned into pHIS-Parallel2 vector using NdeI and XhoI restriction sites by ATG:biosynthetics GmbH (Merzhausen, Germany) (3). Per a 1 consists of two repeats and is highly prone to degradation. Previous work on the homologue Bla g 1 focused on the second repeat only (4), which is why we sub-cloned Per a 1 repeat 2 (residue 197-378) using 5’-GAG ACA TAT GGG TCG CAA TCT GCA A-3’ as forward and 5’-TCT CCT CGA GTC AGA ACA AGC TGC G-3’ as reverse primer. Protein expression was performed in *E. coli* BL21Star at 37°C for 3 hours after induction with 0.4 mM IPTG. Harvested bacteria were dissolved in 10 mM NaOAc pH 8.6, 10 mM EDTA and broken by repeated freeze and thaw cycles. The first purification step involved an anion exchange chromatography using a 5 ml HiTrap DEAE column and the ÄKTA Prime purification system (GE Healthcare). Proteins were eluted with 1 M NaCl buffer and fractions containing enriched Per a 1 were pooled. Buffer exchange to 2 mM Tris pH 8.6 and concentration of the protein sample was achieved using an Amicon Ultra-15 Centrifugal Filter with 3 kDa cut-off (Millipore). Finally, size exclusion chromatography using a Superdex 75 10/300 GL column connected to an ÄKTA FPLC system (GE Healthcare) was performed. Eluting proteins were supplemented with 2 mM PMSF and fractions containing non-tagged Per a 1 repeat 2 were pooled.

**Per a 2**

The mature protein sequence of Per a 2 (Q1M0Y6) was ordered at ATG:biosynthetics (Merzhausen, Germany) and sub-cloned into the pHisParallel2 vector (40) with NdeI and XhoI restriction sites using 5’-GAG ACA TAT GGG TGA TCC AGT CGT CGT-3’ and 5’-TCT CCT CGA GCA GTT CTT CTA CGG A-3’. A glycine was introduced at the N-terminus for enhanced protein stability and the protein was constructed for expression with a C-terminal His_tag_. Protein expression was conducted in *E. coli* SHuffle (Novagen, Gibbstown, NJ, USA). The bacterial culture was induced with 0.4 mM isopropyl-b-D-thiogalactopyranoside (IPTG) and grown at 10°C for 48 hours. Cells were harvested by centrifugation at 4,000 g for 20 min and cells were lysed by three cycles of freezing and thawing followed by sonication in 10 mM sodium phosphate pH 7.0, 1 M NaCl and 6 M urea (lysis buffer). Supernatants after centrifugation at 14.000 g for 30 min were filtered and loaded onto a 1 mL His-Trap HP column (GE Healthcare, Little Chalfont, UK) using the ÄKTA Protein Purification Systems (GE Healthcare). A washing step of 30 ml lysis buffer was performed followed by an on-column refolding gradient of 400 ml to 10 mM sodium phosphate pH 7.0, 1 M NaCl. Proteins were gradient eluted using 10 mM sodium phosphate pH 7.0, 1 M NaCl, 500 mM imidazole.

**Per a 3**

The gene corresponding to Per a 3.0101 (Q25641) was ordered at ATG:biosynthetics (Merzhausen, Germany) and the Per a 3 C-domain harboring the IgE binding epitopes sub-cloned into the pHisParallel2 vector (40) with NdeI and XhoI restriction sites using 5’-GAG ACA TAT GGG TCC CCG TTA C’ and 5’-TCT CCT CGA GCT TGT GGA AGA T-3’. The construct was transformed into *E. coli* Rosetta-gamiB pLysS and expression was induced with 0.4 mM IPTG. Recombinant expression of the C-terminal His_tag_ protein was conducted at 16°C overnight and cell harvest and disruption performed by freeze and thaw cycles (see Per a 1). The bacterial pellet was first dissolved in 10 mM Na_2_HOP_4_ pH 7.4, 250 mM NaCl, 10 mM imidazole and 6 M urea, the resulting pellet after centrifugation was dissolved again in 10 mM Na_2_HOP_4_ pH 7.4, 250 mM NaCl, 10 mM imidazole and 0.5% SDS. The protein extract was loaded on a 5 ml HisTrap FF crude column and elute using 10 mM Na_2_HOP_4_ pH 7.4, 250 mM NaCl, 500 mM imidazole, 0.1% sarkosyl. For refolding purpose and to keep the protein in solution, Per a 3 C-domain was buffer exchanged to 10 mM Na_2_HOP_4_ pH 7.4, 0.1% sarkosyl. For stability in long-time storage, 20 mM EDTA and 10 mM PMSF were added to the final pool.

**Per a 4:**

Per a 4 (AY792948) without signal peptide was generated in *E. coli*. A codon optimized synthetic gene (GeneArt, Thermo Fisher Scientific) was subcloned into expression vector pET11a. The Per a 4/pET11a construct was transformed to Origami2(DE3) *E. coli* cells, which were used for protein expression (3 L in Luria Broth (LB) low salt medium) using isopropyl β-D-1-thiogalactopyranoside (IPTG) to a final concentration of 1 mM, at an optical density (OD_600_) of 0.7 for induction of protein expression over night at 22°C. Cells were harvested by centrifugation (20’, 5000xg) and lysed using 20 mM Bis-Tris (pH 5.8) by three times freeze/thawing cycles under liquid nitrogen. Lysate was prepared by centrifugation (30 min, 13,000xg, 30 min, 4°C) and filtration (5 µm, 0.8 µm, 0.45 µm). For purification of recombinant Per a 4 the lysate was applied to two step anion exchange chromatography (Hi Prep DEAE FF 16/10 followed by HiPrepQ HP 16/10, GE Healthcare, Freiburg, Germany). Protein concentration of Per a 4 was measured using the BCA protein assay (Thermo Fisher Scientific).

**Per a 5**

For cDNA cloning of Per a 5 gene freeze dried cockroach powder was utilized for total RNA isolation using EZ-RNA isolation kit (Biological Industries Sartorius) and converted to cDNA by RevertAid(H-) cDNA synthesis kit (Thermo Fisher Scientific). Per a 5 gene (AY792949.1) was PCR amplified using gene specific primers. The PCR product and vector backbone were cleaved by Eco R I and Xho I, gel eluted by QIAquick gel extraction kit (Qiagen, Germany) and ligated using T4 DNA Ligase (Thermo Fisher Scientific) at 16 ºC overnight. Ligation mixture was transformed in *E.coli* DH5α cells and positive colonies were grown overnight at 37ºC. Restriction digestion of positive colony showed the presence of 651 bp insert. The clone was also confirmed by gene sequencing. Forward primer: 5’-GTA TTG GAA TTC GAT GAC CAT CGA CTT CTA C-3’. Reverse primer: 5’-ACT GAA CTC GAG CTT CTT GGC GAG GTT ATC-3’. Recombinant Per a 5 (rPer a 5) was expressed in *E.coli* BL21-λ DE3 cells. Briefly, the clone containing Per a 5 was grown until OD_600_ reached 0.5 and induced with 1mM IPTG (isopropyl-β-D-thiogalactopyranoside) for 4 h followed by harvesting. The cell pellets obtained were dissolved in binding buffer (50 mM Tris, 300 mM NaCl), sonicated and centrifuged. Meanwhile, Ni-NTA agarose (Qiagen, Germany) resin was equilibrated with binding buffer and incubated with supernatant for 2 h at 4ºC. The column was washed with wash buffer (binding buffer with 25 mM imidazole) and eluted with elution buffer (binding buffer containing 250 mM imidazole). Protein elutes were concentrated and dialyzed with Amicon concentrator (Millipore) and re-purified with Ni-NTA resin. The re-purified elutes were concentrated, estimated (by BCA assay) and resolved as a single band at approximately 27 kDa on 12% SDS-PAGE.

**Per a 7, Per a 8 and Per a 9**: *E. coli* optimized synthetic genes (GeneArt, Thermo Fisher Scientific). for Per a 7.0101 (Y14854), Per a 8 (JQ279816) and Per a 9 (AY563004) were subcloned into expression vector pET23, and transformed into BL21(DE3) *E. coli* cells. Protein expression of 2 L (Per a 7, Per a 9) or 8 L (Per a 8) *E. coli* culture was induced by addition of IPTG to a final concentration of 1 mM, at an optical density (OD_600_) of 0.7. After 16 hours at 22°C, the cultures of Per a 7 and Per a 8 were harvested and the cells lysed in 20 mM Bis-Tris (pH 6) containing 4 M urea for 30 min at room temperature on an end over end rotator (Reax2, Heidolph-Instruments). The recombinant proteins were purified from the supernatant of the lysates by two step anion exchange chromatography (Hi Prep DEAE FF 16/10 followed by HiPrepQ HP 16/10, GE Healthcare) using on column refolding on DEAE column with a 4-0 M urea gradient followed by a 0-1 M NaCl gradient for elution, both in 20 mM Bis-Tris (pH6). Elution fractions containing the recombinant proteins were pooled and applied to size exclusion chromatography (HiLoad 26/600 Superdex 75 pg, GE Healthcare) using 20 mM MOPS, 0.5 mM NaCl (pH 7) as running buffer. For purification of rPer a 9 the cells were lysed in 20 mM Tris (pH 8) by three times freeze/thawing cycles under liquid nitrogen. Lysate was prepared by centrifugation (30 min, 13,000 g, 30 min, 4°C) and filtration (5 µm, 0.8 µm, 0.45 µm). Subsequently the lysate was applied to anion exchange chromatography (MonoQ HR5/5, GE Healthcare, Freiburg, Germany) followed by size exclusion chromatography (HiLoad 26/600 Superdex 75 pg, GE Healthcare) using 20 mM MOPS, 0.5 mM NaCl (pH 7.6) as running buffer as final polishing step. Protein concentration of the recombinant proteins was measured by the BCA protein assay (Thermo Fisher Scientific).

**Per a 10**

For cDNA cloning of Per a 10 total RNA was isolated from freeze‐dried *Periplaneta americana* using EZ‐RNA kit (Biological Industries, Kibbutz Beit‐Haemek, Israel) and cDNA synthesized as per manufacturer's protocol (Qiagen‐Sensiscript RT Kit, Hilden, Germany). cDNA was amplified by gene specific primers SP 1 5‘-GTA TTG GAATTC ATG CTT CGC TAC CTG GTA-3’ and SP 2 5‘-ACT GAA GCT CGA GGT TAG TTG ACT CAG TCT GTT CTG-3’ with Eco RI and Xho 1 sites, respectively, at flanking ends. PCR conditions include initial denaturation at 95°C for 5 min and then 30 cycles of 94°C 1 min, 55°C 1 min, 72°C 1 min, with final extension at 72°C for 7 min. PCR product and pET22b+ vector were digested with Eco RI and Xho 1 restriction enzymes, ligated, then transformed into *E. coli* DH5α cells and positive clones were confirmed by restriction digestion. The clone was sequenced at The Centre for Genomic Application, Delhi and designated as pSP10. For recombinant expression of rPer a 10 *E. coli* BL21 cells having pSP10 were grown until optical density 0.6, induced with 1 mM isopropyl β‐D‐1‐thiogalactopyranoside (Fluka, Sigma, St. Louis, MO, USA) for 4 h and cells harvested at 7600 RCF for 10 min at 4°C. The cell pellet was resuspended in binding buffer (50 mM Tris, 300 mM NaCl and 10 mM imidazole; pH 8.0), sonicated and centrifuged for 30 min at 4°C. The supernatant was incubated with 1 mL Ni‐NTA resin (Qiagen) for 1 h at 4°C and washed with 20 mM imidazole in binding buffer. His_tag_ protein was eluted using 250 mM imidazole, desalted and concentrated using Amicon Ultra‐15 (Millipore, Billerica, MA, USA). Recombinant Per a 10 (rPer a 10) amount was estimated (by BCA assay) and polyacrylamide gel electrophoresis performed under non‐reducing conditions to determine the homogeneity (5).

**Physicochemical characterization of *Periplaneta* allergens**

For consistent analyses, all purified recombinant proteins were re-buffered to 10 mM of potassium phosphate pH 7.0 using centrifugal filter devices (Ultra-Amicon) (Millipore corporation, Billerica, US), by several rounds of centrifugation at 12.000 rpm.

**Amino acid analysis**

Amino acid analysis (AAA) was performed with all proteins in duplicates following the Pico-Tag method (Waters, Milford, MA, USA). Phenylthiocarbamyl amino acid derivatives were analyzed by reversed phase high-performance liquid chromatography (UltiMate 3000, Thermo Fischer Scientific, Waltham, MA, USA), using a 3.0x150 mm XSELECT™ HSS T3 3.5 µm column (Waters). Hydrolyzed amino acids were quantified at 254 nm by peak area comparison to the amino acid standard H (Pierce, Rockford, IL, USA).

**SDS-PAGE analysis**

Based on the protein concentration obtained by AAA, 2 µg of all recombinant allergens were loaded on 15% polyacrylamide gels under reducing conditions. Proteins were visualized with Coomassie Brilliant Blue R-250 staining (Bio-Rad, Hercules, CA, USA). Images were taken with ChemiDoc MP Imaging System (Bio RAD) and acquired with the software Image Lab. 4.0.1.

**Mass spectrometry analysis**

For intact mass measurements of purified allergens, the samples were desalted with C_18_ ZipTips (Merck, Millipore, Billerica, MA, USA) and directly infused into the Q-Exactive mass spectrometer (Thermo Fisher Scientific) at a flow rate of 1 µL/min, using the nano-electrospray head. Raw data obtained from intact proteins were processed with Protein Deconvolution 2.0 (Thermo Fisher Scientific). Additionally, intact mass measurements were performed with reduced and alkylated proteins. The samples were reduced and alkylated with reducing and blocking buffer from the ProteoExtract All-in-One Trypsin Digestion Kit (Merck Millipore, Billerica, USA) and measured as described above. For peptide mapping analysis 1 µg of each protein was reduced, alkylated and digested with the mentioned Trypsin Digestion Kit. Resulting peptides were separated by reverse-phase nano-HPLC (Dionex Ultimate 3000, Thermo Fisher Scientific). Peptides were loaded onto the trap column (PepSwift Monolithic Trap Column, Dionex) and desalted with 0.1% (v/v) heptafluorobutyric acid at a flow rate of 10 µL/min. After 5 min, trap and separation column (PepSwift Monolithic Nano Column, 100 µm x 25 cm, Dionex) were coupled with a switching valve and the peptides were eluted with an acetonitrile gradient (Solvent A: 0.1% (v/v) Formic acid (FA), 0.01% (v/v) Trifluoroacetic acid (TFA), 5% (v/v) Acetonitrile (ACN); solvent B: 0.1% (v/v) FA, 0.01% (v/v) TFA, 90% (v/v) ACN; 5–45% B in 60 min) at flow rate of 1 µL/min at 55°C. The HPLC was directly coupled via nano-electrospray to a Q-Exactive Orbitrap mass spectrometer (Thermo Fisher Scientific). Capillary voltage was 2 kV. For peptide identification, a top 12 method was used. The normalized fragmentation energy was 28%. Survey and fragment spectra were analyzed with Proteome Discoverer version 1.4 (Thermo Fisher Scientific) or Peaks Studio 7.5 (Bioinformatics Solutions, Waterloo, Canada), respectively.

**Circular dichroism and dynamic light scattering**

Circular dichroism spectra to study the secondary structure of all purified allergens were recorded in 10 mM of potassium phosphate pH 7.0 with a JASCO J-815 spectropolarimeter (Jasco, Tokyo, Japan). Far UV spectra (190 - 260 nm) were baseline subtracted and results are presented as mean residue molar ellipticity. Three-dimensional structure model of all allergens were generated using SWISS-MODEL (<https://swissmodel.expasy.org/>). The global model quality estimate (GMQE) was considered for the quality of the models. Three-dimensional structure representations were generated with Chimera 1.8 (www.cgl.ucsf.edu/chimera).

The aggregation behavior of the proteins was analyzed by dynamic light scattering (DLS). DLS analyses were performed with protein concentrations of 1 mg/mL and the data acquisition (10 measurements of 5 seconds duration) was done using DLS802 system (Viscotek Corp). Three independent measurements were performed for each protein and the results are presented as mean of three experiments.

**Figure legends**

Table S1: Identity and physicochemical characterization of recombinant *Periplaneta* allergens.

Table S2: *Periplaneta-*, *Blattella* extract and recombinant *Periplaneta* allergen specific ImmunoCAP values of 117 patients enrolled in the study (I: India; II: Korea; III: Venezuela; IV: Iran). Cut-off 0.1 kU_A_/L, except for * (cut-off 0.35 kU_A_/L).

Fig. S1: Secondary structure analysis of *Periplaneta* allergens. Circular dichroism spectra (190 – 260 nm) of purified allergens measured at 20°C. Three-dimensional structure models generated with SWISS-MODEL and presented with Chimera 1.8.

Fig. S2: Dynamic light scattering analyses of recombinant *Periplaneta* allergens. Rh, hydrodynamic radius in nm.

Fig. S3: Identification of Per a 4 and Per a 8 by MS/MS analysis of *Periplaneta* extracts (protein extraction was performed twice). Number and identity of peptides, as well as % sequence coverage and potential amino acid modification are indicated.

Fig. S4: Comparison of IgE antibody levels to *Periplaneta* (i206) and *Blattella* (i6) in the respective patient collectives from India (n=35), Korea (n=27), Venezuela (n=25) and Iran (n=30).

Fig. S5: CRD of cockroach allergy by experimental ImmunoCAP testing in country subgroup of patients (India: n=29, Korea: n=27, Venezuela: n=22 and Iran: n=20) preselected by positive *Periplaneta* or *Blattella*-specific ImmunoCAP values. *Periplaneta* (i206) and *Blattella* (i6) extract, and *Periplaneta* allergen specific IgE values for each patient are depicted. Cut-off 0.1 kU_A_/L, except for * (cut-off 0.35 kU_A_/L).

REFERENCES

1. Sudha VT, Arora N, Gaur SN, Pasha S, Singh BP. Identification of a serine protease as a major allergen (Per a 10) of Periplaneta americana. *Allergy* (2008) **63**:768–76. doi:10.1111/j.1398-9995.2007.01602.x

2. Sudha VT, Srivastava D, Arora N, Gaur SN, Singh BP. Stability of protease-rich periplaneta Americana allergen extract during storage: formulating preservatives to enhance shelf life. *Journal of clinical immunology* (2007) **27**:294–301. doi:10.1007/s10875-007-9078-y

3. Sheffield P, Garrard S, Derewenda Z. Overcoming expression and purification problems of RhoGDI using a family of "parallel" expression vectors. *Protein expression and purification* (1999) **15**:34–9. doi:10.1006/prep.1998.1003

4. Mueller GA, Pedersen LC, Lih FB, Glesner J, Moon AF, Chapman MD, et al. The novel structure of the cockroach allergen Bla g 1 has implications for allergenicity and exposure assessment. *J Allergy Clin Immunol* (2013) **132**:1420–6. doi:10.1016/j.jaci.2013.06.014

5. Goel C, Govindaraj D, Singh BP, Farooque A, Kalra N, Arora N. Serine protease Per a 10 from Periplaneta americana bias dendritic cells towards type 2 by upregulating CD86 and low IL-12 secretions. *Clinical & Experimental Allergy* (2012) **42**:412–22. doi:10.1111/j.1365-2222.2011.03937.x
